# Astragaloside IV Alleviates the Experimental DSS-Induced Colitis by Remodeling Macrophage Polarization Through STAT Signaling

**DOI:** 10.3389/fimmu.2021.740565

**Published:** 2021-09-13

**Authors:** Lianlian Tian, Jun-Long Zhao, Jian-Qin Kang, Shi-bo Guo, Nini Zhang, Lei Shang, Ya-Long Zhang, Jian Zhang, Xun Jiang, Yan Lin

**Affiliations:** ^1^Department of Pediatrics, Tangdu Hospital, Air Force Medical University, Xi’an, China; ^2^State Key Laboratory of Cancer Biology, Department of Medical Genetics and Developmental Biology, Air Force Medical University, Xi’an, China; ^3^Department of Health Statistics and Ministry of Education, Key Laboratory of Hazard Assessment and Control in Special Operational Environment, Air Force Medical University, Xi’an, China; ^4^State Key Laboratory of Cancer Biology, Department of Biochemistry and Molecular Biology, Air Force Medical University, Xi’an, China

**Keywords:** inflammatory bowel disease, astragaloside IV (AS-IV), macrophages polarization, STAT signaling, pro-resolving macrophage

## Abstract

Inflammatory bowel disease (IBD) is characterized by chronic and relapsing intestinal inflammation, which currently lacks safe and effective medicine. Some previous studies indicated that Astragaloside IV (AS-IV), a natural saponin extracted from the traditional Chinese medicine herb *Ligusticum chuanxiong*, alleviates the experimental colitis symptoms *in vitro* and *in vivo*. However, the mechanism of AS-IV on IBD remains unclear. Accumulating evidence suggests that M2-polarized intestinal macrophages play a pivotal role in IBD progression. Here, we found that AS-IV attenuated clinical activity of DSS-induced colitis that mimics human IBD and resulted in the phenotypic transition of macrophages from immature pro-inflammatory macrophages to mature pro-resolving macrophages. *In vitro*, the phenotype changes of macrophages were observed by qRT-PCR after bone marrow-derived macrophages (BMDMs) were induced to M1/M2 and incubated with AS-IV, respectively. In addition, AS-IV was effective in inhibiting pro-inflammatory macrophages and promoting the pro-resolving macrophages to ameliorate experimental colitis *via* the regulation of the STAT signaling pathway. Hence, we propose that AS-IV can ameliorate experimental colitis partially by modulating macrophage phenotype by remodeling the STAT signaling, which seems to have an essential function in the ability of AS-IV to alleviate the pathological progress of IBD.

## Introduction

Inflammatory bowel disease (IBD) includes two distinct disorders, Crohn’s disease (CD) and ulcerative colitis (UC), which is a complex disease caused by the interaction of environmental factors, immune disorders, and bacterial imbalances in the genetic background (1–3). The incidence of IBD is rising in the world, especially in Africa and Asia (4, 5). Multiple factors, such as genetic background, environmental changes, abnormal gut microbiota, and mucosal immune dysregulation, have been suggested to contribute to IBD pathogenesis. The current therapies for IBD mainly include anti-inflammatory drugs and biologics, such as corticosteroids, 5-aminosalicylic acid (5-ASA)-based agents, azathioprine, and TNF-α inhibitors (6, 7). However, the side effects of these medicines were unacceptable to some people, such as nausea, diarrhea, and abdominal pain. More important, even with an aggressive top-down approach to therapy, the majority of patients fail to achieve prolonged, steroid-free remission and are at particular risk of requiring surgery. Therefore, it is crucial to devise novel therapeutic strategies for the treatment of IBD.

The previous studies have demonstrated that mucosal immunity plays a significant role in establishing and maintaining gut homeostasis, especially for intestinal macrophages. During inflammation, the terminal differentiation of monocytes and maturation of intestinal macrophages are disrupted (8, 9). At the onset of intestinal inflammation, accumulated immature macrophages maintain their pro-inflammatory ability by secreting inflammatory cytokines, such as IL-12, IL-23, and IL-1β, which is similar to the classic macrophage activation pattern (also known as M1). With the development of IBD, phagocytosis of apoptotic cells triggers the functional transition of macrophages to the pro-resolving phenotype (10, 11), similar to the alternative macrophage activation pattern (also known as M2). The pro-resolved intestinal macrophages increase anti-inflammatory cytokines production, repress pro-inflammatory factors secretion, enhance phagocytic activity, and acquire the expression of scavenger receptors that are essential for homeostasis (12). It is significant for IBD recovery to remodel mucosal immunity and facilitate pro-resolving macrophages development.

Traditional herbs have been used to treat colitis in China for hundreds of years (13) and have been increasingly recognized worldwide for their low toxicity, low side effects, and is well tolerated. Of note, AS-IV (3-O-β-D-xylopyranosyl-6-O-β-D-glucopyranosyl cycloastragenol), a natural saponin isolated from *Astragali radix*, has been reported to present antioxidant, cardioprotective, anti-inflammatory, antiviral, antibacterial, antifibrosis, anti-diabetes, and immunoregulation effects (14, 15). In addition, AS-IV could alleviate experimental colitis symptoms *in vitro* and *in vivo* (16, 17). However, the definite mechanisms of AS-IV remain unclear. Recent studies have shown that AS-IV enhances diabetic wound healing involving upregulation of alternatively activated macrophages (18). In addition, AS-IV inhibits progression and metastasis of lung cancer by regulating macrophage polarization through the AMPK signal (19). AS-IV also has been demonstrated to ameliorate steroid-induced osteonecrosis of the femoral head by repolarizing the phenotype of pro-inflammatory macrophages (20). However, during IBD progression, whether and how AS-IV exerts pro-resolving effects *via* remodeling intestinal macrophages development remains to be explored. In this study, we reported that AS-IV attenuated clinical activity of DSS-induced colitis and resulted in the phenotypic transition of macrophages. Further mechanism studies revealed that AS-IV ameliorates experimental IBD partially by modulating macrophage phenotype *via* the regulation of the STAT signaling pathway.

## Materials and Methods

### Animals

Male specific-pathogen-free (SPF) C57BL/6 J mice (6-8 weeks old, 21 ± 2 g) were purchased from Experimental Animal Center, Air Force Medical University (Xi’an, China). They acclimated for 7 days with tap water and a pelleted basal diet before the start of the experiments. The temperature was maintained at 23°C ± 2°C, humidity 50%-60%, 12h light/dark cycles. Throughout the experiments, mice were fed with a standard chow diet and tap water. The animal room was cleaned regularly during the holding period.

### DSS-Induced Colitis

To induce experimental colitis, mice were induced in the animals *via* oral administration of 2.5% (w/v) DSS (MW 36,000–50,000; MP Biomedicals) in fresh drinking water for 5 consecutive days. For evaluation of colitis progression, mice received normal drinking water for additional 10 days. AS-IV powders (C41H68O14, molecular weight: 784.9702, purity >98%) (Dalian Meilun) was dissolved in 0.5% sodium carboxymethyl cellulose (CMC) at a concentration of 10mg/mL or 20mg/mL before experiment. The mice were orally administered 50 or 100 mg/kg AS-IV once daily for 10 days (from day 0 to day 10) and 5-Aminosalicylic acid(5-ASA) 150 mg/kg was used as the positive control (Med Chem Express Co.). The body weight of the mice was monitored every day. The disease activity index (DAI) score was determined as previously reported (21). In the termination of the study, the mice were euthanized using CO_2_, and the colon was removed from animals. The length of the colon was measured. All experimental procedures were approved by the Ethical Committee of Air Force Medical University.

### Histological Analysis

The distal section of colon tissues was immediately fixed in 4% formalin overnight at room temperature, embedded in paraffin wax, and stained with H&E. The histological score of the colon was determined as previously described (22).

### Measurement of MPO

The distribution number of neutrophils in colonic samples was detected by Myeloperoxidase (MPO) activity. Colonic samples were weighed and homogenized using reaction buffer. Then, the MPO activity assay kit (Nanjing Jiancheng Co.) was applied to assess the MPO activity.

### Real-Time PCR Assay (RT-PCR)

After obtaining murine lamina propria immune cells or BMDMs, Trizol reagent (Invitrogen) was used to extract total RNA immediately. NanoDrop 2000/2000c UV-Vis (Thermo scientific, USA) was used to measure RNA quantification. We also assessed the contamination by the A260/280, and the integrity by electrophoresis to confirm RNA quality which including purity and integrity. cDNA was synthesized by using 1μg of total RNA with a reverse transcription kit (Takara, Dalian, China) following the supplier’s instructions that means the reaction was run under the conditions of 37°C for 15 minutes and 85°C for 5 seconds. 250 nM of specific primers, a kit (SYBR Premix EX Taq, Takara) and the ABI Prism 7500 Real-Time PCR System were used for quantitative real-time PCR in triplicates under the conditions of step one (95°C for 15 minutes) and 40 cycles of step two (one cycle: 95°C for 10 seconds, 58°C for 20 seconds and 72°C for 20 seconds), with β-actin as an internal control. The gene expression level was normalized by subtracting the expression level of β-actin of the same group, and the different expression levels were calculated using the comparative Ct (2-^△△^Ct) method. Primers are listed in **Table S1** in **Supplementary Material**.

### Cell Culture and AS-IV Treatment

Bone marrow cells were harvested from tibias and femurs of C57BL/6J mice and subjected to erythrolysis by using red blood cell lysis buffer. Then, the cells were cultured at a density of 2 × 10^6^/mL in DMEM containing 10% FBS and 25 ng/mL murine macrophage-colony stimulating factor (M-CSF) (PeproTech, Rocky Hill, NJ) for 7 days to obtain BM-derived macrophages (BMDMs). BMDMs were stimulated with IFNγ (20 ng/ml, SinoBiology, China) and LPS (50 ng/mL, Sigma, Saint Louis, MO) or IL-4 (20 ng/mL, SinoBiology) for 24 h before further analyses. Macrophages treated with PBS, LPS + IFN-γ, or IL-4 were named M^PBS^, M^LPS^, or M^IL-4^, according to Epelmann et al. (23).

AS-IV was dissolved in dimethyl sulfoxide (DMSO) for the treatment of macrophages. The final concentration of DMSO was less than 0.1% (v/v). For treatment with AS-IV (100μM), cells were incubated for 24 h after BMDM treated with PBS, LPS + IFN-γ, or IL-4. In some experiments, phosphorylated STAT1 inhibitors (Med Chem Express Co.) were added and treated with AS-IV (100μM) after BMDM treated with PBS, LPS + IFN-γ, or IL-4. To exclude the effects of DMSO, the adherent macrophages were treated with DMSO (0.1%) alone for 24 h.

### Isolation of Murine Lamina Propria Immune Cells From Colonic Tissues

Isolation of colonic lamina propria cells was performed according to the protocol. Entire colons from each group were longitudinally cut and washed to remove feces. They were then cut into 1 cm pieces, followed by incubation with a predigestion solution containing 5 mM EDTA and 0.145 mg/ml DTT for 20 min at 37°C on a shaking platform. After removal of EDTA by three washes in PBS and passing through a cell strainer (100 µm), the suspension of epithelial, subepithelial, and villus cells was removed. The remaining colon pieces, including lamina propria cells and muscle layer, were cut by using scissors and then incubated in digestion media containing 0.05 g of collagenase D (Roche), 0.05 g of DNase I (Sigma-Aldrich), and 0.3 g of dispase II (Roche) for 25 min at 37°C on a shaking platform. After digestion, the lamina propria cells were enriched using Percoll density gradient centrifugation. The resulting cells were then used for flow cytometry analysis.

### Western Blot Analysis

BMDMs and colonic lamina propria cells were harvested and the whole-cell lysates were extracted on ice with the RIPA buffer containing a protease inhibitor cocktail (Beyotime, Haimen, China). Lysates were centrifuged and the supernatants were collected. Protein concentration was determined using a BCA protein assay kit (Pierce). Aliquots of protein lysates were separated by 10% SDS-PAGE and blotted onto polyvinylidene fluoride membrane. Membranes were blocked with bovine serum albumin solution and then incubated overnight at 4°C with a 1:1000 dilution of STAT1, p-STAT1, STAT3, p-STAT3, and β-Actin antibodies, followed by HRP-conjugated secondary antibodies. Protein bands were visualized with chemiluminescent reagents (Pierce).

### Flow Cytometry

Colonic lamina propria cells were stained with antibodies against CD45, CD11b, F4/80, MHC II, or Ly6C for 30 min at 4°C in the dark, then washed twice and resuspended in 500μL of phosphate-buffered saline (PBS). The cells were sorted on a FACS AriaIII flow cytometer (BD Immunocytometry Systems) and analyzed using the FlowJo software (Ashland, OR).

### Luciferase Assay

A DNA fragment containing 4 × STAT1 recognized elements were synthesized and inserted into pGL3-promoter to construct a pGL3-STAT1 reporter, which is used to monitor the activation of STAT1 protein. HeLa cells (2 × 10^4^) were transfected with pGL3-basic and pGL3-STAT1 reporters using Lipofectamine 2000™ (Invitrogen), TNF-α, and AS-IV of different concentrations were added simultaneously. The luciferase activity was assessed 48 h later using Luminoskan Ascent (Labsystems, Helsinki, Finland) and a Dual-Luciferase Reporter Assay Kit (Promega) according to the manufacturer’s protocol. All luciferase activity was normalized to the Renilla luciferase activity.

### Immunostaining and Confocal Microscopy

Colon tissues were removed, rinsed in PBS, embedded in O.C.T. compound (Sakura Finetek), and frozen in an isopentane bath on dry ice. Sections (5 μm) were cut on a microtome and were stained with a mixture of antibodies, including an anti-Rabbit CD206 and anti-Rat F4/80. The slides were counterstained with DAPI (Vector Laboratories, Inc.) to identify nuclei. Images were observed and captured with mages were captured with an FV1200 laser scanning confocal microscope (Olympus, Inc.). The positive area was quantified using Viewer software CaseViewer and analyzing five high power fields per section and per animal.

### Homology Modeling and Molecular Docking

STAT1 protein sequences were downloaded from the UniProt database (https://www.uniprot.org/), then submitted sequences to the Robetta server (http://robetta.bakerlab.org/). Homology modeling of protein structure was performed using the TrRefineRosetta modeling method. Further, the protein structure was submitted to the website (https://saves.mbi.ucla.edu/) for Ramachandran plot analysis. The results showed that the Ramachandran plot score was 92.4%, which indicated that the structure of the protein was more accurate and could be used for the subsequent molecular docking study. The docking of AS-IV with STAT1 was performed with the Autodock 4.1 software. Firstly, the protein structure was set by hydrogenation and charge additional. Then the energy of the small molecule compound ligand (AS-IV) was minimized, and rotatable bonds and the numbers were set. The docking pocket coordinates were x: 25.0, y: 34.0, and z: -32.0, with a radius of 20 Å. Finally, the molecular docking was evaluated according to the docking energy, and the optimal docking conformation was retained to demonstrate a 2-dimensional diagram.

### Statistical Analysis

Statistical analysis was performed with the Graph Pad Prism 8.0 software. Student’s t-test or one-way ANOVA test was used for statistical analyses. All the experiments were performed at least three times, and the acquired results are shown as mean ± SD. P<0.05 was considered statistically significant.

## Results

### Immature Macrophages Recruitment Facilitated the Inflammatory Progression of IBD

**Figure 1:**
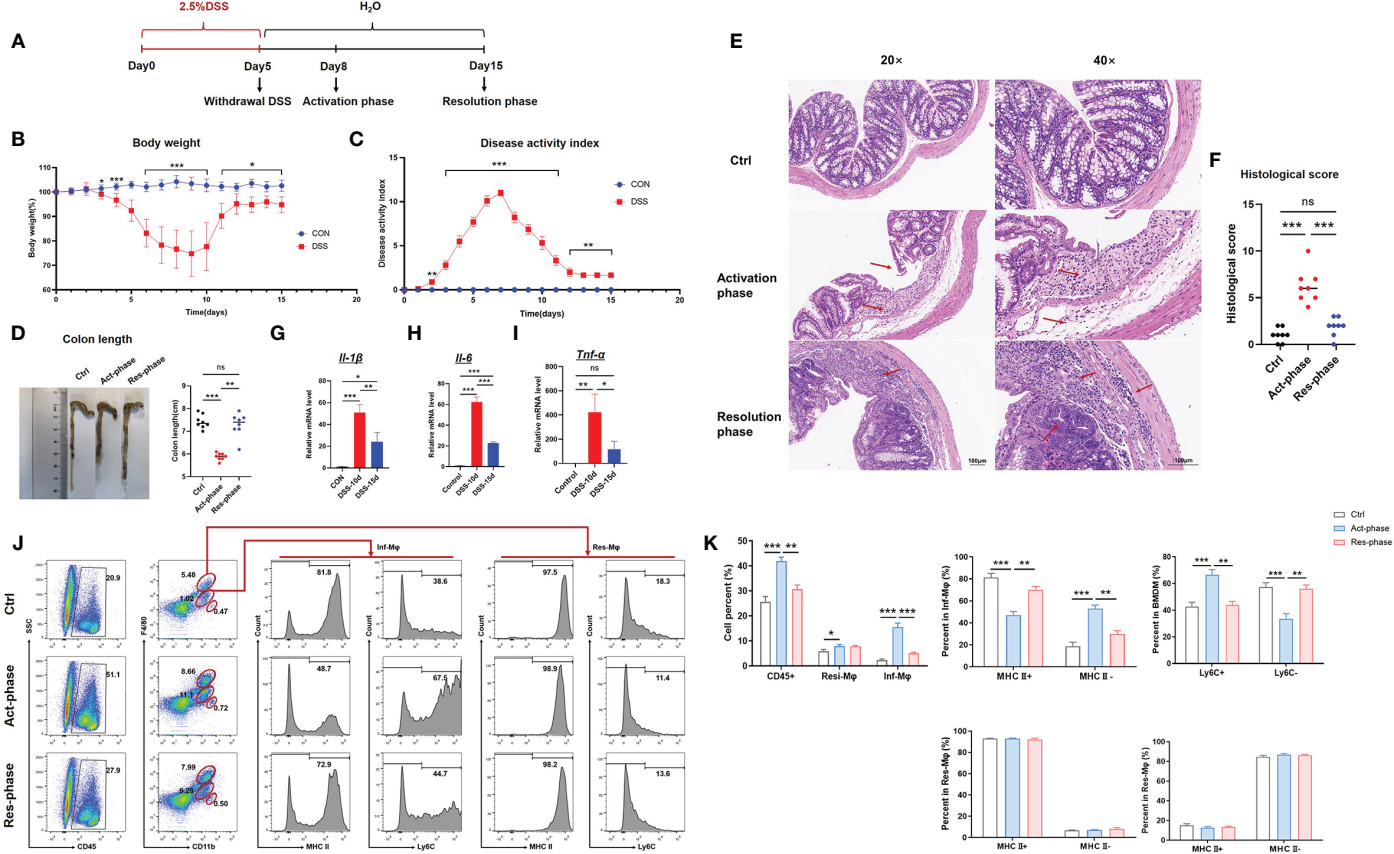
Immature macrophages recruitment facilitated the inflammatory progression of IBD. **(A)** Establishment of DSS-induced colitis mice. **(B)** The daily mean weight change in the activation phase and resolution phase of DSS-induced colitis. **(C)** The changes in DAI in different stage phases scored from diarrhea, bleeding, and body weight loss. **(D)** On the activation phase (Act-phase) and resolution phase (Res-phase), the mice were sacrificed, their colons were removed, and the lengths of their colons were measured and recorded. Macroscopic appearance of the colon, as represented by the colon with the mean colon length. **(E)** The histopathological changes in the colon tissue samples were examined by H&E staining (20×, 40×). **(F)** Histopathological scores were determined for the colon tissue samples. **(G–I)** The relative mRNA expression levels of the inflammatory cytokines *Il-1β*, *Il-6*, and *Il-10* in colon tissue were determined by RT-PCR. **(J)** Intestinal macrophages were analyzed by fluorescence-activated cell sorting (FACS) after staining with anti-F4/80, anti-CD11b, and anti-MHCII (or anti-Ly6C). **(K)** The number of the different subpopulations in **(J)** was determined and quantitatively compared. The student’s t-test or One-way ANOVA test was used for statistical analyses. Bars represent means ± SD; ^*^ *P < *0.05, ^**^P < 0.01, ^***^ *P < *0.001. ns, no significance.

IBD is featured by a series of events comprising an activation phase and gradually followed by a resolution phase (12, 24). To assess the changes of inflammation in experimental IBD mice at different phases, we established the DSS-induced colitis model (**Figure 1A**). The mice treated with DSS presented significant presentations of IBD, including weight loss, bloody stool, and diarrhea. The weight loss observed in the DSS group was significantly aggravated from the 7th day after DSS treatment and began to regain weight from the 10th day (**Figure 1B**). The results of the DAI score showed that the gross colitis of DSS-induced mice appears and gradually worsened from the 3rd day of modeling (**Figure 1C**). Colon length was further measured and statistically processed. Compared with the control group, the results showed that colon length was shortened in the activation phase and increased in the resolution phase (**Figure 1D**). The changes of mice’s intestinal mucosa in the acute active phase group were observed using HE staining. The results showed specific pathological changes of IBD, including epithelial cell shedding, crypt structure destruction, infiltration of lymphocytes and neutrophils, connective tissue hyperplasia, and intestinal gland dilatation. However, intestinal inflammation gradually returned to homeostasis in the resolution phase (**Figure 1E**). The histopathological score were significantly higher in the activation phase group than in the control group. Compared with the activation phase group, the histopathological scores of the resolution phase group decreased and were close to the control group (**Figure 1F**).

Most steady-state mouse colonic macrophages are derived from a CC-chemokine receptor 2 (CCR2)-dependent infiltration of lymphocyte antigen 6C-high (Ly6C^hi^) blood-derived monocytes. When Ly6C^hi^ monocytes enter the lamina propria, they undergo a gradual differentiation and acquire expression of major histocompatibility complex II (MHCII) whereas loss expression of Ly6C. This phenotypic differentiation of blood-derived monocytes is paralleled by a progressive acquisition of typical functions of mature pro-resolving macrophages (8, 25). Therefore, we evaluated the development of immune cells by using FACS. The results indicated that the inflammatory macrophages recruited (CD11b^+^F4/80^low^, Inf-Mφ) from bone marrow were increased significantly at the activation phase (DSS 10 days) and retrieved at the resolution phase (DSS 15 days), while the resident macrophages (CD11b^+^F4/80^hi^, Res-Mφ) presented no difference. More importantly, the promotion of inf-Mφ was majorly contributed to the immature macrophage population with MHCII^-^Ly6C^+^ phenotype (**Figures 1J, K**). It has been reported that MHCII^-^Ly6C^+^ macrophages were identified with M1-like polarization and presented pro-inflammatory functions (12). Therefore, we further determined the relative mRNA levels of inflammatory cytokines, including *Il-1β*, *Tnf-α*, and *Il-6*. The results suggested that the expression of these cytokines was increased at the activation phase and reduced at the resolution phase (**Figures 1G–I**). Taken together, these data demonstrated that the recruitment of MHCII^-^Ly6C^+^ immature macrophages promoted the inflammatory response and facilitated the damage of IBD.

### AS-IV Attenuates Inflammatory Progression of DSS-Induced Colitis

**Figure 2:**
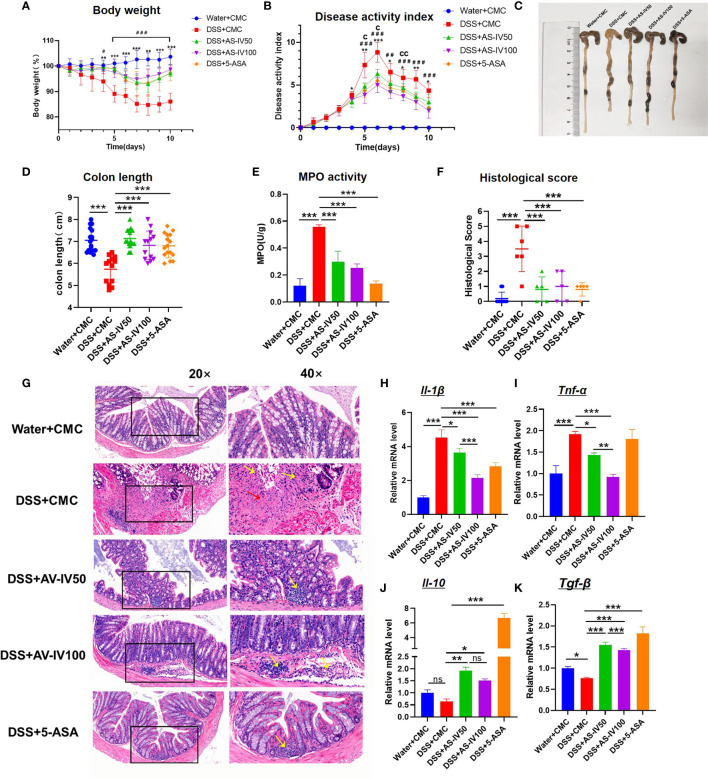
AS-IV treatment attenuates inflammatory progression of DSS-induced colitis mice. The following groups of mice were used in the study: Ctrl (Water+CMC); Model group (DSS+CMC); DSS+AS-IV50mg/kg (DSS+AS-IV50); DSS+AS-IV100mg/kg (DSS+AS-IV100); and 5-ASA 150 mg/kg treatment(5-ASA) (n=8), and each group was used for three independent experiments. The mean values ± SEMs are represented by bars. **(A)** The daily mean weight change in each group (*P < 0.05, **P < 0.01, ***P < 0.001.DSS+AS-IV50 *versus* DSS+CMC). (^#^P < 0.05, ^##^P < 0.01, ^###^P < 0.001.DSS+AS-IV100 *versus* DSS+CMC). **(B)** The changes in DAI, scored from diarrhea, bleeding, and body weight loss (^c^P < 0.05, ^cc^P < 0.05 DSS+AS-IV100 *versus* DSS+AS-IV50). **(C)** On day 10, the mice were sacrificed, their colons were removed, and the lengths of their colons were measured and recorded. **(D)** Macroscopic appearance of the colon, as represented by the colon with the mean colon length. **(E)** Colonic MPO activity. **(F)** Histopathological scores were determined for the colon tissue samples. **(G)** The histopathological changes in the colon tissue samples were examined by H&E staining (20×, 40×). **(H–K)** The relative mRNA expression levels of the inflammatory cytokines *Il-1β*, *Tnf-α*, *Il-10*, and *Tgf-β* in colon tissue were determined by RT-PCR, Student’s t-test or One-way ANOVA test was used for statistical analyses. Bars represent means ± SD; ^*^ *P <*0.05, ^**^ P <0.01, ^***^ *P <*0.001. ns, no significance.

At present, IBD is recurrent and lacks effective medicine (26). Some previous studies demonstrated that AS-IV alleviates the symptoms of multiple inflammatory diseases (15). To characterize the effect of AS-IV on the evolution of IBD, we established the DSS-induced colitis model with AS-IV administration. Meanwhile, the treatment of 5-ASA, which is identified as the routine medicine for IBD, was considered as the positive control. After AS-IV treatment, bodyweight loss in the colitis model was significantly alleviated and similar to the Ctrl group and 5-ASA group (**Figure 2A**). Additionally, the DSS/AS-IV50 group and DSS/AS-IV100 group exhibited a dose-dependently decreased cumulative DAI compared with the DSS+CMC group (**Figure 2B**). Furthermore, the reduction in colon length, which is a marker of intestinal inflammation, was less pronounced in DSS/AS-IV50 group and DSS/AS-IV100 group compared with the DSS group (**Figures 2C, D**). As expected, MPO activity, an indicator of neutrophil aggregation, was significantly reduced in the AS-IV treatment group than in the DSS group (**Figure 2E**). Additionally, H&E histopathology results showed that treatment with AS-IV significantly alleviated the inflammatory response in comparison with treatment with the DSS group (**Figure 2G**). Compared with the DSS group, the blinded histological injury scores in the distal colon of the AS-IV treatment group were significantly decreased (**Figure 2F**). Previous studies showed certain cytokines and pro-inflammatory mediators were increased in DSS-induced colitis. Therefore, we further investigated whether the protective effects of AS-IV on DSS-induced colitis in mice were correlated with reductions in pro-inflammatory mediator and cytokine production. The results indicated that the expression of *Il-1β* and *Tnf-α* mRNA was significantly increased in the colon of the DSS group, whereas AS-IV could substantially decrease the expression of *Tnf-α* and *Il-1β* in a dose-dependently manner (**Figures 2H, I**). Meanwhile, AS-IV could dramatically increase the expression of *Il-10* and *Tgf-β* in a dose-dependently way (**Figures 2J, K**). These results indicated that AS-IV attenuates the inflammatory progression in DSS-induced colitis *in vivo*.

### AS-IV Remodeled the Development of Intestinal Macrophages

**Figure 3:**
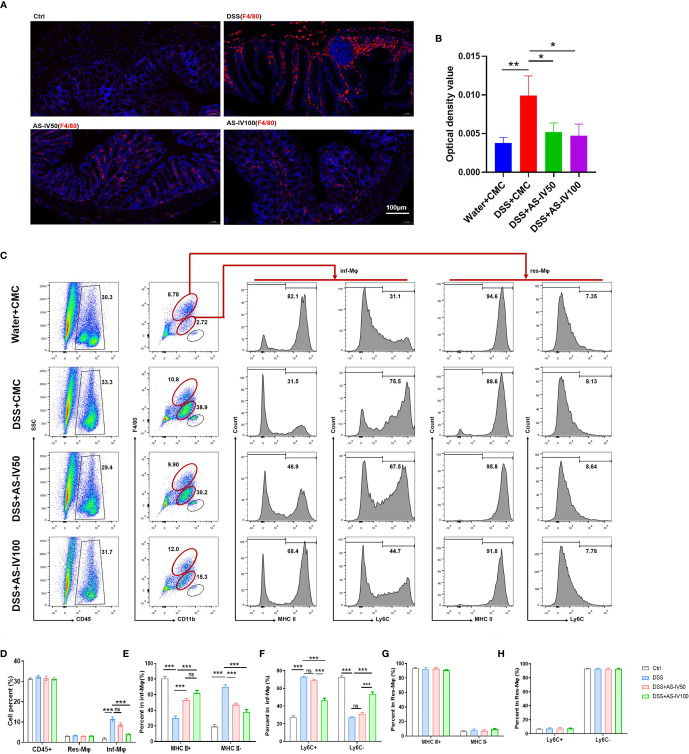
AS-IV remodeled the development of intestinal macrophages. **(A)** F4/80 protein expression in colon tissue was detected by immunofluorescence. Positive immunoreactivity for F4/80 protein expression is indicated by the red color. The slides were counterstained with DAPI (blue). In addition, the sum of OD was analyzed in **(B). (C)** Intestinal macrophages were collected and analyzed by fluorescence-activated cell sorting (FACS) after staining with anti-F4/80, anti-CD11b, and anti-MHCII (or anti-Ly6C). **(D–H)** The number of the different subpopulations in **(C)** was determined and quantitatively compared. One-way ANOVA test was used for statistical analyses. Bars represent means ± SD; ^*^ *P < *0.05, ^**^P < 0.01, ^***^ *P < *0.001. ns, no significance.

As mentioned above, macrophages are the gatekeepers of intestinal immune homeostasis. Decreasing intestinal macrophage leads to a loss of tolerance to symbiotic bacteria and food antigens, which is thought to be the foundation for the chronic inflammation observed in IBD (27). Therefore, immunofluorescence staining was performed to evaluate the number of infiltrated macrophages. The results suggested that DSS-induced promotion of macrophage was retrieved by AS-IV administration (**Figures 3A, B**). In addition, different subpopulations of intestinal macrophages play different roles during IBD progression. The immature macrophage with inflammatory functions was increased significantly at the activation phase of IBD (**Figure 1J**). We detected the effect of AS-IV on the development of intestinal macrophages by FACS (**Figure 3C**). The results indicated that the increase of Inf-Mφs induced by DSS stimulation was restored by AS-IV administration, although Res-Mφs were not influenced. Moreover, AS-IV significantly promoted MHCII^lo^Ly6C^hi^ immature macrophages to differentiate into MHCII^hi^Ly6C^lo^ mature macrophages with resolving functions (**Figures 3D–H**). Furthermore, the number of infiltrated granulocytes (CD45^+^CD11b^+^F4/80^hi^) was also reduced by AS-IV treatment (**Figure 3C**). Taken together, these findings indicated that AS-IV improved the immune microenvironment *via* remodeling intestinal myeloid cells development and protected from DSS-induced colitis.

### AS-IV Administration Modulated the Plasticity of Intestinal Macrophages

**Figure 4:**
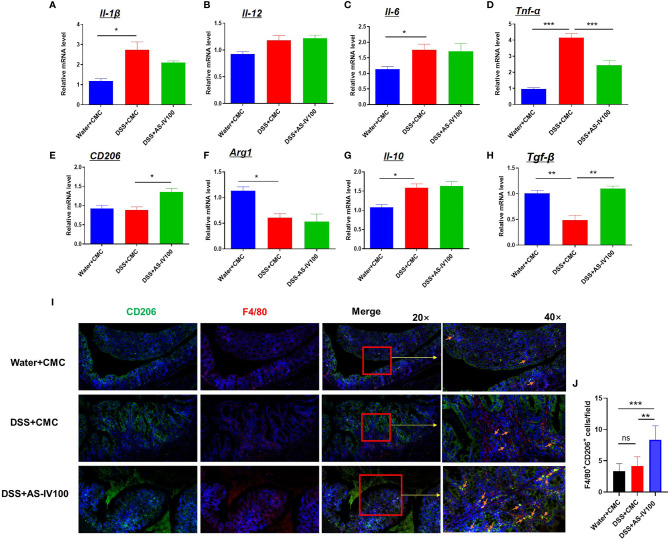
AS-IV administration modulated the plasticity of intestinal macrophages. **(A–H)** Intestinal macrophages were collected with F4/80 maker by flow cytometric sorting. The relative mRNA expression levels of the inflammatory cytokines *Il-1β*, *Il-12*, *Il-6*, *Tnf-α*, *CD206*, *Arg1*, *Il-10*, and *Tgf-β* in F4/80 positive intestinal macrophages were determined by RT-PCR. The mean values ± SEMs are represented by bars. **(I)** The protein expression levels of the F4/80 (red color) and CD206 (green color) in colon tissue by immunofluorescence, and the slides were counterstained with DAPI (blue). **(J)** The number of F4/80^+^CD206^+^ cells in **(I)** were counted and quantitatively compared. One-way ANOVA test was used for statistical analyses. Bars represent means ± SD; ^*^ *P < *0.05, ^**^P < 0.01, ^***^ *P < *0.001. ns, no significance.

Previous studies have shown that macrophages exhibit a distinct phenotype and functional differences in response to different inflammatory stimuli, manifested as M1/M2 polarized macrophages. However, the phenotype of a macrophage is difficult to accurately define as M1 or M2 polarization in many diseases. The plasticity of intestinal macrophages would be altered during the progression of IBD (12, 27). To assess the effect of AS-IV on macrophages polarization, we sorted intestinal macrophages by flow cytometric and examined the mRNA expression levels of polarization markers and cytokines by RT-PCR, including *Il-12*, *Il-1β*, *Il-6*, *Tnf-α*, *CD206*, *Arg1*, *Il-10*, and *Tgf-β*. As presentation in **Figures 4A–D**, DSS-induced increases in the mRNA expression levels of the pro-inflammatory cytokines *Tnf-α* were decreased by AS-IV treatment. In contrast, mRNA expression levels of the pro-resolving cytokines *CD206* and *Tgf-β* were increased by AS-IV treatment compared with the DSS group (**Figures 4E–H**). Additionally, we examined the protein expression levels of the F4/80 and CD206 in colon tissue by immunofluorescence, which represented pro-resolving macrophages (**Figures 4I–J**). Consistent with the above results, immunofluorescence results showed that pro-resolving macrophages were significantly increased by treatment with AS-IV. Taken together, these results indicated that AS-IV had protective roles against DSS-induced colitis mediated by the phenotypic transformation of macrophages from pro-inflammatory macrophages to pro-resolving macrophages.

### AS-IV Promoted Transformation of Macrophage Subsets *In Vitro*

**Figure 5:**
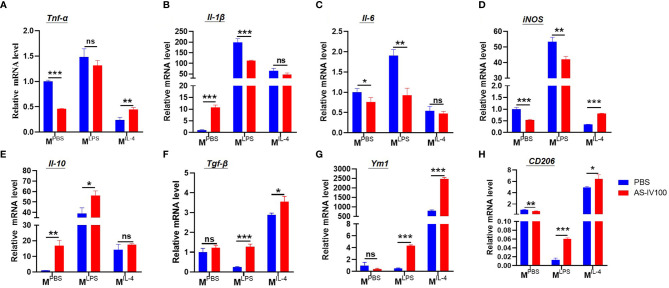
AS-IV promoted transformation of macrophage subsets *in vitro* **(A–H)** Bone marrow-derived macrophages (BMDMs) were stimulated with PBS (M^PBS^), LPS + IFN-γ (M^LPS^), or IL-4 (M^IL-4^) following treatment with AS-IV. The relative mRNA expressions of the M1-related genes *Tnf-α*, *IL-1β*, *Il-6*, *iNOS*, and the expression of M2-related genes, including *Il-10*, *Tgf-β*, *Ym1*, *CD206* were determined by RT-PCR. Student’s t-test was used for statistical analyses. Bars represent means ± SD; ^*^ *P < *0.05, ^**^P < 0.01, ^***^ *P < *0.001. ns, no significance.

To further study whether the therapeutic effect of AS-IV on DSS-induced colitis leads to changes in macrophage phenotypes. Firstly, BMDMs were cultured and then stimulated with PBS (M^PBS^), LPS + IFN-γ (M^LPS^), or IL-4 (M^IL-4^) following treatment with AS-IV, and the expression of polarization markers was detected by qRT-PCR. Then we examined the expression of M1-related genes, including *Tnf-α*, *Il-1β*, *Il-6*, *iNOS*, and the expression of M2-related genes, including *Il-10*, *Tgf-β*, *Ym1*, *CD206* in the colon. The results showed that AS-IV downregulated the expression of M1 markers *Il-1β*, *Il-6*, *iNOS* under LPS+IFN-γ stimulation. Whereas the expressions of M2 marker *Tgf-β*, *Ym1*, *CD206* were upregulated under IL-4 stimulation (**Figures 5A–H**). Taken together, these results indicated that AS-IV significantly suppresses pro-inflammatory macrophage subsets and promotes pro-resolving macrophage subsets *in vitro*.

### AS-IV Regulated Macrophages Phenotype Through STAT1 Signaling

There are several signals, including NF-κB, JAK-STATs, and IRFs, which play regulatory roles in the development, functional regulation, and subpopulation transformation of macrophages (28, 29). These pathways form a complex regulatory network through close interaction and crosstalk. Among them, STAT signaling cascades are vital regulators of the differentiation and function of the macrophage (30).

**Figure 6:**
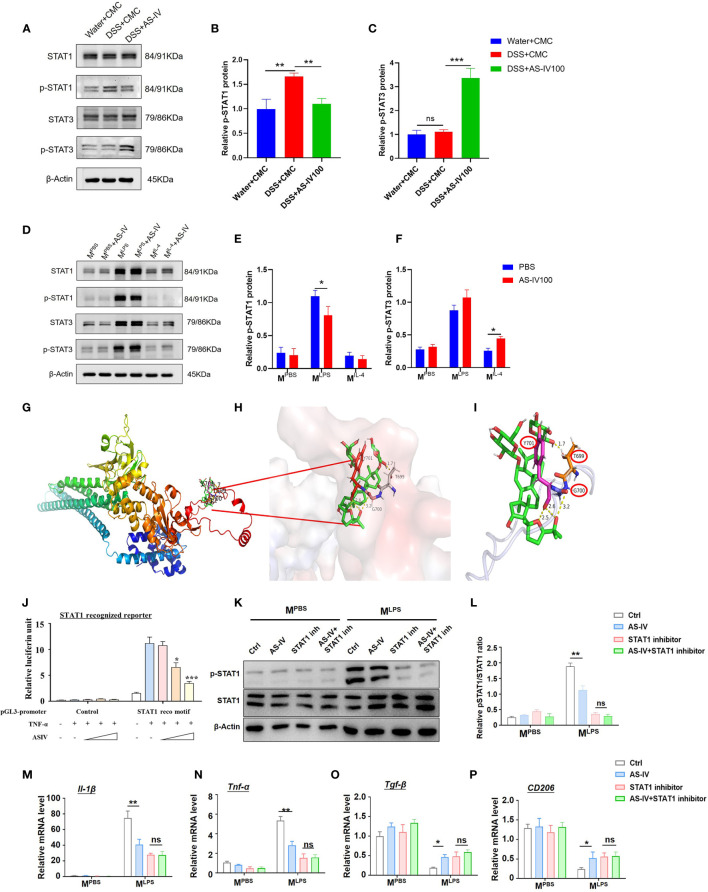
AS-IV regulated macrophages phenotype through STAT1 signaling. **(A–C)** Western blotting analysis of STAT1, p-STAT1, STAT3, and p-STAT3 protein expression in mice colon tissue. **(D–F)** Western blotting analysis of STAT1, p-STAT1, STAT3, and p-STAT3 protein expression after BMDMs were stimulated with PBS (M^PBS^), LPS + IFN-γ (M^LPS^), or IL-4 (M^IL-4^) following treatment with AS-IV. **(G–I)** Homology modeling and molecular docking were used to analyze the interaction between STAT1 and AS-IV. **(J)** HeLa cells were transiently transfected with STAT1 recognized reporters, TNF-α and AS-IV of different concentrations were added simultaneously. The luciferase activity was assessed 48 h later. **(K–L)** STAT1 was inhibited in M^PBS^/M^LPS^ by STAT1 inhibitor, followed by AS-IV treatment, and then the expression of p-STAT1and STAT1were detected by western blotting analysis. **(M–P)** STAT1 was inhibited in M^PBS^/M^LPS^ by STAT1 inhibitor, followed by AS-IV treatment, and then the expression of *Il-1β*, *Tnf-α*, *CD206*, and *Tgf-β* was detected by RT-PCR. student’s t-test or One-way ANOVA test was used for statistical analyses. Bars represent means ± SD; ^*^ *P < *0.05, ^**^P < 0.01, ^***^ *P < *0.001. ns, no significance.

Previous studies showed dysfunction of and genetic variation in JAK-STAT signaling pathways correlated with a spectrum of IBD (31). Therefore, we focused on the STAT signaling pathway and evaluated STAT signaling activity after different stimulation in the presence or absence of AS-IV in DSS-induced colitis in mice. Western blot analysis indicated that p-STAT1 was down-regulated with AS-IV treatment, while p-STAT3 was up-regulated with AS-IV treatment (**Figures 6A–C**). We further evaluated STAT signaling activity in the presence or absence of AS-IV in the different phenotypes of BMDM. The results showed that the expression of p-STAT1 and p-STAT3 in M^LPS^ macrophages was significantly higher than that in M^PBS^ and M^IL-4^ macrophages. Meanwhile, western blot analysis similarly indicated that p-STAT1 was down-regulated with AS-IV treatment and p-STAT3 was up-regulated with AS-IV treatment especially in LPS stimuli (**Figures 6D–F**).

It has been reported that multiple polyhydroxy glycosides regulated the phosphorylation of different proteins (32, 33). Chemically speaking, polyhydroxy substrates induce the transfer of phosphate groups from phosphorylated proteins. However, this phosphorylation transfer requires the spatial proximity of phosphorylated protein to the polyhydroxy molecule. Therefore, to investigate the mechanisms of AS-IV regulating STATs phosphorylation, the molecular docking simulation was carried out. The results indicated that AS-IV could bind with a pocket of STAT1 consisting of 699-701 aa (**Figures 6G–I**). Meanwhile, we constructed the reporter system with the STAT1 recognition motif, which could evaluate the activation of the STAT1 pathway by the intensity of luciferin. The reporter assay indicated that the activity of the STAT1 pathway was activated by TNF-α stimulating immensely, while was repressed by AS-IV administration in a dosage-dependent manner (**Figure 6J**). The results demonstrated that AS-IV interacted with STAT1 and mediated the dephosphorylation and deactivation of STAT1. Meanwhile, western blot analysis further indicated that p-STAT1 was down-regulated with AS-IV treatment in M^LPS^ macrophages, while inhibition of p-STAT1 completely canceled the effect of AS-IV on macrophage in LPS stimuli **(** **Figures 6K, L** **)**. In addition, STAT1 was inhibited in M^PBS^/M^LPS^ by p-STAT inhibitor, followed by AS-IV treatment; and then the expression of macrophage polarization markers was detected. The results suggested that inhibition of p-STAT1 completely canceled the effect of AS-IV on macrophage polarization switch, indicating that the function of AS-IV was dependent on the regulation of STAT1 signaling **(** **Figures 6M–P** **)**. Considering that STAT1 signaling could repress the activation of the STAT3 pathway, AS-IV might regulate STAT3 signaling through repressing STAT1 activation. The data above indicated that AS-IV regulates macrophage function *via* repressing the STAT1 signaling pathway.

## Discussion

IBD, including UC and CD, is a chronic and relapsing intestinal inflammation with symptoms involving acute abdominal pain, chronic diarrhea, and loss of body weight. The pathogenesis of the disease is still unclear, and the available therapeutic strategies for the disease are not up to the researcher’s expectations. Several compounds extracted from Chinese herbs have been shown to have therapeutic effects against UC (13). AS-IV is a lanolin alcohol-shaped tetracyclic triterpenoid saponin as one of the major active substances of *Astragalus membranaceu* (15). Recent studies have found that AS-IV regulates energy metabolism and further improves 2,4,6-trinitrobenzene sulfonic acid (TNBS)-induced colitis (16). Additionally, AS-IV has a therapeutic effect on experimental UC *in vitro* and *in vivo via* inhibiting inflammatory molecules and downregulating NF-κ B signaling (17). Our studies also showed that AS-IV attenuated the DAI and MPO activity, downregulate the expression of *Tnf-α* and *Il-1β* and increase the level of *Il-10* and *Tgf-β* in a dose-dependently manner in DSS-induced colitis mice. Although these studies have pointed to that AS-IV alleviates the symptoms of experimental ulcerative colitis, the target cells of AS-IV acting in the intestine have yet to be fully elucidated.

Intestinal macrophages restrain excessive inflammation responded to harmless commensal microorganisms and improve tolerance mainly *via* the production of Il-10 (34). Therefore, intestinal macrophages play pivotal roles in establishing and maintaining gut homeostasis. However, recent genome-wide association studies identified key driver genes of macrophages for inflammatory disorders (35). When intestinal homeostasis is disturbed, the composition of the intestinal macrophage pool will change significantly (36). Therefore, we first observed that the immature Inf-Mφs population recruited from bone marrow were increased significantly at the activation phase and retrieved at the resolution phase. While the number of Inf-Mφs decreased after the administration of AS-IV at the activation phase. Immunofluorescence validated the conclusion that AS-IV decreased macrophage percentages in DSS-induced colitis mice. Previous studies have indicated that macrophages show great phenotypic and functional differences in response to different external inflammatory stimulus signals, which are manifested as M1/M2 macrophages. However, M1/M2 macrophage classification is largely based on *in vitro* differentiation, which does not accurately reflex the complexity of *in vivo* macrophage plasticity and heterogeneity (37). Current evidence suggests that monocytes that have entered tissues differentiate into macrophages displaying varying M1-like or M2-like characteristics (38, 39). Accumulation of M2-like pro-resolving macrophages in the intestinal microenvironment appears to play a vital role in re-establishing gut tissue homeostasis (40, 41). Further, we studied the effect of AS-IV on the phenotype and function of intestinal macrophages. In this study, we observed that AS-IV administration resulted in the phenotypic transformation of macrophages from pro-inflammatory M1 (M^LPS^) macrophages to pro-resolving M2 (M^IL-4^) macrophages. Meanwhile, flow cytometry results showed AS-IV treatment could reduce the number of bone marrow-derived proinflammatory macrophages and promote their functional maturation.

The activation of stimulus-specific transcription factors within this macrophage-specific transcriptional landscape is probably to dictate the polarization of macrophages, such as STAT family, nuclear receptor PPARγ, CREB–C/EBP axis, and interferon regulatory factors (42). JAK-STAT signaling is an important signaling pathway associated with a spectrum of hematological malignancies and autoimmune disorders, such as IBD (43). In addition, JAK-STAT signaling mediated essential cytokines (IL-6, IL-10, IL-2 or IL-22) participating in immune and stromal gut cell homeostasis and those well-described mediators (IFN-γ, IL-12, IL-23 or IL-9) involving in pathological processes in IBD (31, 44). Several small-molecule JAK inhibitors have shown efficacy in the treatment of IBD within the past 5 years (45). However, JAK activation leads to structure alteration, which triggers a series of subsequent modifications and ultimately results in the phosphorylation, dimerization, and activation of the STATs family. While a number of studies have indicated that STATs are crucial factors in macrophage polarization and involved in IBD progression (46, 47). Particularly, p-STAT1 has the potent effect on promoting M1 type macrophage activation in the presence of IFN-γ (48). Studies have reported amelioration of experimental colitis has been observed in STAT1-deficient mice (49), suggesting has a pro-inflammatory effect. However, regulatory and/or anti-inflammatory properties have also been described for STAT1 in intestinal epithelial cells or macrophages (50, 51). In this study, we observed that p-STAT1 significantly upregulated in the DSS group, while decreased with AS-IV treatment. But p-STAT3 was up-regulated with AS-IV treatment. STAT3 is a key immunomodulatory transcription factor that has a fundamental role in IBD (43, 52). Previous studies showed STAT3 has an antagonistic effect on immunostimulatory effects of STAT1 and imbalance of STAT1/3 signals is the pathogenesis of various inflammatory diseases (53, 54). Our results showed AS-IV treatment seems to regulate the balance between p-STAT1/3. The mechanism investigation demonstrated that AS-IV, as a ligand, specifically interacts with STAT1, mediating the dephosphorylation of Tyr701 and deactivation of STAT1. Meanwhile, many studies indicated that STAT1 signaling could repress the activation of the STAT3 pathway. Therefore, we inferred that AS-IV directly repressed STAT1 signaling, and subsequently activated STAT3 signaling. Further, we examined the effects of AS-IV on STAT signaling during macrophage polarization *in vitro*. The protein expression of p-STAT1 was significantly decreased in M^LPS^ macrophages treated with AS-IV, while p-STAT3 was increased in M^LPS^ macrophages treated with AS-IV. Further reporter assay showed that STAT1 pathway was repressed by AS-IV administration with a dosage-dependent manner directly. Finally, we confirmed that the AS-IV treatment canceled the regulation of macrophages phenotype after inhibiting STAT1 signal by salvage experiment. Taken together, these data demonstrated that AS-IV may regulate macrophages phenotype through STAT1 signaling.

In conclusion, we studied the therapeutic effects and mechanism of AS-IV on experimental colitis and provided compelling evidence that AS-IV was effective in IBD treatment. Our findings showed that AS-IV attenuated clinical disease activity, diminished pro-inflammatory cytokine production, up-regulated anti-inflammatory cytokine production, and decreased the percentages of macrophages by blocking the M1 polarization of macrophages partially through the STAT1 signaling pathway in DSS-induced colitis. Taken together, these findings indicate that transition of intestinal macrophage subsets is a likely therapeutic target in IBD therapy and provide new insights regarding the therapeutic potential of AS-IV for IBD treatment.

## Data Availability Statement

The original contributions presented in the study are included in the article/**Supplementary Material**. Further inquiries can be directed to the corresponding authors.

## Ethics Statement 

The animal study was reviewed and approved by the Ethical Committee of Air Force Medical University, China.

## Author Contributions 

YL, XJ, JZ, and J-LZ designed the study and wrote the manuscript. LT, S-BG, Y-LZ, and J-LZ performed experiments. YL, LT, N-NZ, LS, and JQ-K analyzed data. YL supervised the work and edited the manuscript. All authors contributed to the article and approved the submitted version.

## Funding

This work was supported by the National Natural Science Foundation of China (82000522, 81802841) and the China Postdoctoral Science Foundation (2021M693944).

## Conflict of Interest

The authors declare that the research was conducted in the absence of any commercial or financial relationships that could be construed as a potential conflict of interest.

## Publisher’s Note

All claims expressed in this article are solely those of the authors and do not necessarily represent those of their affiliated organizations, or those of the publisher, the editors and the reviewers. Any product that may be evaluated in this article, or claim that may be made by its manufacturer, is not guaranteed or endorsed by the publisher.
