# Supplementary material for: Astragaloside IV Alleviates the Experimental DSS-Induced Colitis by Remodeling Macrophage Polarization Through STAT Signaling
Source: Front Immunol. 2021 Sep 13;12:740565. doi: 10.3389/fimmu.2021.740565 (PMC8473681; doi:10.3389/fimmu.2021.740565)
Supplement: Supplementary file 1 [file Table_1.docx]

**Supplemental Tables S1**

**Table S1.** Primers used for PCR.

| Gene | Forward Primer | Reverse Primer |
| --- | --- | --- |
| *Il-1β* | 5′-TAGACAACTGCACTACAGGCTCCGA-3′ | 5′-GGGTCCGACAGCACGAGGCT-3′ |
| *Il-6* | 5′-CTGCAAGAGACTTCCATCCAG-3′ | 5′- AGTGGTATAGACAGGTCTGTTGG -3′ |
| *Il-10* | 5′-CCAAGCCTTATCGGAAATGA-3′ | 5′- TTTTCACAGGGGAGAAATCG -3′ |
| *Il-12* | 5′-GGAAGCACGGCAGCAGAATA-3′ | 5′-AACTTGAGGGAGAAGTAGGAATGG-3′ |
| *iNOS* | 5′-GCAGAGATTGGAGGCCTTGTG-3′ | 5′-GGGTTGTTGCTGAACTTCCAGTC-3′ |
| *Tgf-β* | 5′-GGAGGTACCGCCCGGCCCGC-3′ | 5′-GACAGCAATGGGGTTCGGG -3′ |
| *Tnf-α* | 5′-ATGAGCACAGAAAGCATGATC-3′ | 5′-TACAGGCTTGTCACTCGAATT-3′ |
| *Ym1* | 5′-CAGGTCTGGCAATTCTTCTGAA-3′ | 5′-GTCTTGCTCATGTGTGTAAGTGA-3′ |
| *Arg-1* | 5'-AGACAGCAGAGGAGGTGAAGAG-3′ | 5'-CGAAGCAAGCCAAGGTTAAAGC-3′ |
| *CD206* | 5′‐GTGGAGTGATGGAACCCCAG‐3′ | 5′‐CTGTCCGCCCAGTATCCATC‐3′ |
| *β-Actin* | 5′-TTCCTTCTTGGGTATGGAAT-3′ | 5′-GAGCAATGATCTTGATCCTC-3′ |

| Gene | GenBank Accession | Forward Primer Length | Reverse Primer Length |
| --- | --- | --- | --- |
| *Il-1β* | [NM_008361](http://www.ncbi.nlm.nih.gov/entrez/query.fcgi?cmd=Search&db=Nucleotide&term=NM_008361) | 25 | 20 |
| *Il-6* | [NM_031168](http://www.ncbi.nlm.nih.gov/entrez/query.fcgi?cmd=Search&db=Nucleotide&term=NM_031168) | 21 | 23 |
| *Il-10* | [NM_010548](http://www.ncbi.nlm.nih.gov/entrez/query.fcgi?cmd=Search&db=Nucleotide&term=NM_010548) | 20 | 20 |
| *Il-12* | [NM_001159424](http://www.ncbi.nlm.nih.gov/entrez/query.fcgi?cmd=Search&db=Nucleotide&term=NM_001159424) | 20 | 24 |
| *iNOS* | [NM_010927](http://www.ncbi.nlm.nih.gov/entrez/query.fcgi?cmd=Search&db=Nucleotide&term=NM_010927) | 21 | 23 |
| *Tgf-β* | [NM_011577](http://www.ncbi.nlm.nih.gov/entrez/query.fcgi?cmd=Search&db=Nucleotide&term=NM_011577) | 20 | 19 |
| *Tnf-α* | [NM_013693](http://www.ncbi.nlm.nih.gov/entrez/query.fcgi?cmd=Search&db=Nucleotide&term=NM_013693) | 21 | 21 |
| *Ym1* | 12655(NCBI GeneID) | 22 | 23 |
| *Arg-1* | [NM_007482](http://www.ncbi.nlm.nih.gov/entrez/query.fcgi?cmd=Search&db=Nucleotide&term=NM_007482) | 22 | 22 |
| *CD206* | [NM_008625](http://www.ncbi.nlm.nih.gov/entrez/query.fcgi?cmd=Search&db=Nucleotide&term=NM_008625) | 20 | 20 |
| *β-actin* | [NM_007393](http://www.ncbi.nlm.nih.gov/entrez/query.fcgi?cmd=Search&db=Nucleotide&term=NM_007393) | 20 | 20 |
